# Effectiveness of Epoxy Coating Modified with Yttrium Oxide Loaded with Imidazole on the Corrosion Protection of Steel

**DOI:** 10.3390/nano11092291

**Published:** 2021-09-03

**Authors:** Muddasir Nawaz, Nazal Naeem, Ramazan Kahraman, M. F. Montemor, W. Haider, R. A. Shakoor

**Affiliations:** 1 Center for Advanced Materials (CAM), Qatar University, Doha 2713, Qatar; m.nawaz@qu.edu.qa (M.N.); nazalnaeem14@gmail.com (N.N.); haide1w@cmich.edu (W.H.); 2 Department of Chemical Engineering, Qatar University, Doha 2713, Qatar; 3 Centro de Química Estrutural, Departamento de Engenharia Química, Instituto Superior Técnico, Universidade de Lisboa, Av Rovisco Pais, 1049-001 Lisboa, Portugal; mfmontemor@tecnico.ulisboa.pt; 4 Mechanical and Materials Engineering, Central Michigan University, Mount Pleasant, MI 48859, USA

**Keywords:** epoxy, yttrium oxide, imidazole, coating, corrosion protection

## Abstract

The search for highly effective corrosion protection solutions to avoid degradation of the metallic parts is enabling the development of polymeric organic coatings. Of particular relevance, polymeric nanocomposite coatings, modified with corrosion inhibitors, have been developed to provide enhanced surface protection. In this work, yttrium oxide nanoparticles loaded with corrosion inhibitor (Imidazole), used as additives in the formulation of epoxy for coated on the steel substrate. The loading of Y_2_O_3_ with imidazole was confirmed by field emission scanning electron microscopy (FE-SEM) and Fourier transform infrared spectroscopy (FTIR), thermogravimetric analysis (TGA) and Brunauer–Emmett–Teller analysis. UV-Vis analysis demonstrated the pH-sensitive behavior of the imidazole that helps in self-release when necessary. Electrochemical impedance spectroscopy (EIS) of the coated samples revealed that the coating modified with Y_2_O_3_/IMD provides better corrosion protection compared to coatings containing only Y_2_O_3_. XPS analysis validated the presence of an imidazole protective film on the steel substrate that enhanced the corrosion resistance of the coated samples

## 1. Introduction

Corrosion is a relevant degradation phenomenon in metallic components that affects many industries, leading to high economic burden and safety hazards. A very effective route to protect metallic assets from corrosion is by the application of protective organic coatings. In the last decades, there has been an increase in academic and industrial research in developing organic coatings containing anti-corrosive pigments for the protection of metallic parts in different environments [1,2]. Traditionally, coatings are modified with anti-corrosive pigments that release inhibiting species able to protect the metallic parts, contributing to a higher lifespan [3,4,5]. However, it is important to ensure that these pigments are environmentally compliant. For instance, chromate pigments have been intensively used for a very long time, but chromium (VI) possesses a carcinogenic and toxic nature. Its use has been strongly restricted or even prohibited [6,7]. Among the new non-toxic anticorrosive pigments introduced to substitute chromates, zinc phosphate has been widely accepted since its toxic level is reduced, and many studies have shown good its corrosion inhibition properties [8].

To enhance the corrosion protective efficiency of organic coatings for a longer lifespan, with a low impact on the environment, the use of nanotechnologies to develop carriers for loading active species has opened up a wide range of possibilities in the field [9,10]. The direct addition of corrosion inhibitors into coatings formulation may provide good efficiency but usually, this may lead to unwanted reactions and degradation of the coating matrix which causes early leaching of the inhibitors and decreases the barrier properties of the coating [11,12]. This drawback has been circumvented thanks to the use of carriers loaded with the inhibitors that also helps to enhance the barrier properties of the coating [13,14]. This strategy also helps to prevent unnecessary leaching of inhibitors and achieve more economical and efficient use of the corrosion inhibitors. Notably, the use of carriers requires specific triggers that shall promote the release of inhibitors under certain conditions such as pH change, light or mechanical damage [15]. Various types of carriers, loaded with different actives species, have been studied in recent years, such as metal oxides, layered double hydroxides and layer by layer assemblies based on polyelectrolytes [16,17]. Effective release of inhibitors has been achieved by developing shell-like capsules or porous matrices that are activated under certain conditions. Despite effectiveness, it has been reported that the release of corrosion inhibitors through shell-like capsules is controlled by permeation through the shell material. This may lead to capsules collapse and poor mechanical stability and faster release and early loss of inhibitors. To ensure a stable release of inhibitors when required and for a longer life without loss of mechanical stability, the use of porous inorganic carriers has been proposed as an alternative [18,19].

Therefore, mesoporous oxide nanoparticles (e.g., silica, ceria, and zirconia) have been reported as suitable carriers to transport the corrosion inhibitors [20,21,22]. Yttrium oxide can also be used as a corrosion inhibitor carrier due to its porous character [23]. Yttrium oxide because of its high thermal, chemical, and mechanical stability and low toxicity in nature has been widely used for many applications, including those requiring high corrosion resistance [24]. Therefore, in this work, porous yttrium oxide is selected as carrier for the storage of a corrosion inhibitor.

The azole family, including thiazoles, triazoles and imidazole, has been proposed as corrosion inhibitors for copper, but has been scarcely explored to inhibit steel corrosion. Interestingly, imidazole can be loaded into Y_2_O_3_ and used as anti-corrosive pigments in protective epoxy coatings exposed to aggressive environment [25,26]. Thus, this work aims to investigate the effectiveness of the new anti-corrosive pigment based on yttrium oxide (Y_2_O_3_) loaded with imidazole as a corrosion inhibitor when incorporated into the epoxy formulation for the coating of steel plates. The results revealed that Y_2_O_3_ loaded with imidazole improves the corrosion resistivity of the epoxy coated steel substrates by attaining 99% corrosion inhibition efficiency.

## 2. Materials and Methods

### 2.1. Materials and Chemicals

Yttrium oxide nanoparticles and imidazole as corrosion inhibitor were purchased from Sigma-Aldrich. Epoxy (EPON RESIN 815C) with (EPIKURE 3234) as its curing agent were purchased from Hexion chemicals. Plain carbon steel coupons were purchased from a local supplier with a composition of (Fe = Balance, C = 0.21%, Mn = 0.30%, Cu = 0.20%, S = 0.04%, P = 0.04%). The steel substrate was polished with emery papers from 80 to 320 grit size. After that, rinsed with distilled water and acetone and coupons 30 × 30 × 1.0 mm^3^ were used.

### 2.2. Loading of Corrosion Inhibitor into Y_2_O_3_ Nanoparticles

**Figure 1:**
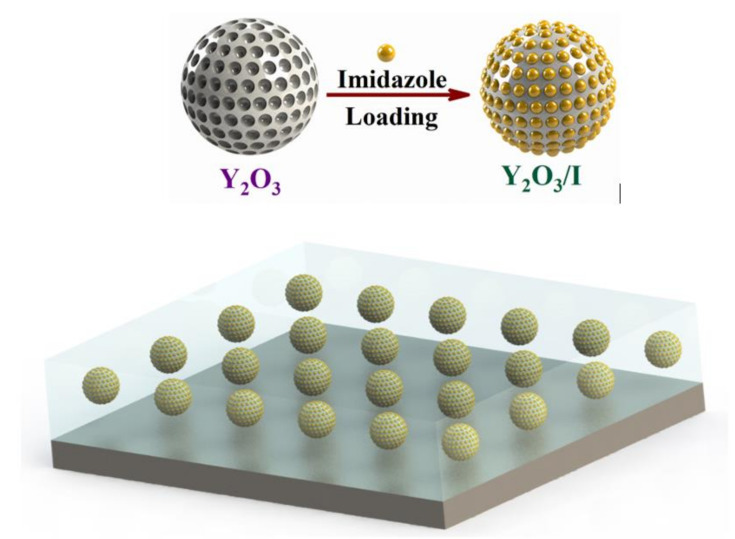
Schematic diagram showing the incorporation of Y_2_O_3_/IMD in the epoxy coating.

Yttrium oxide nanoparticles have a high surface area and are porous. Thus, 3.0 wt. % of Y_2_O_3_ nanoparticles were soaked into 10 mg/mL IMD water solution and sonicated for 30 min at room temperature. After that, the solution was put on a hot plate at 70 °C for 24 h with moderate stirring. The dried loaded Y_2_O_3_ nanoparticles were obtained once the excess solvent removed. The Figure 1 presents the schematic diagram of loading of Y_2_O_3_ with imidazole.

### 2.3. Coating Formulation of Steel Substrate

The 1 wt. % of yttrium oxide loaded with imidazole (Y_2_O_3_/IMD) was added into the epoxy resin with the curing agent and ultrasonicated for the 10 min to obtain a homogeneous mixture. Epoxy was a low viscosity liquid bisphenol A based resin containing a commercial grade of n-butyl glycidyl ether and curing agent unmodified aliphatic amines, while the epoxy to curing agent ratio was kept 4:1. Doctor blade was used for coating on the steel coupons. The dried coating thickness of 100 ± 5 µm was obtained after a compete curing at room temperature for a week.

The adhesion strength of the epoxy coating was 6.4 ± 2 MPa. The FE-SEM cross sectional image for thickness of coating shown in (Figure S1). Two types of coatings were formulated.

i. Reference coating (contained only Y_2_O_3_);
ii. Modified coating (contained Y_2_O_3_/IMD).

### 2.4. Characterization of Nanoparticles and Coating Samples

The phase purity of Y_2_O_3_ and loaded product (Y_2_O_3_/IMD) was investigated by X-ray diffraction (XRD) by using PAN analytical X’pert Pro Cu (Kα), with a scanning rate of 2°/min and scanning angle ranging between 20° ≤ 2θ ≤ 80°. The chemical composition and loading of Y_2_O_3_ nanoparticles were confirmed by FTIR analysis using the FT-IR Frontier (PerkinElmer, Frontier, Waltham, MA, USA) instrument. The spectra were recorded in the range of 4000 to 500 cm^−1^. The surface morphology of the Y_2_O_3_ and Y_2_O_3_/IMD was studied by a field emission scanning electron microscope (FE-SEM-Nova Nano-450, The Netherland) and transmission electron microscopy (TEM, FEI, TALOS F200X, New York, NY, USA). A TGA synchronization analyzer (PerkinElmer, TGA 4000, Boston, MA, USA) was used to analyze the thermal stability of the Y_2_O_3_ and Y_2_O_3_/IMD in the temperature range 30 °C to 600 °C employing a heating rate of 10 °C/minute. The surface charge of Y_2_O_3_ and Y_2_O_3_/IMD was determined by employing Zeta potential equipment (Malvern, Zeta sizer, Nano ZSP, Worcestershire, UK). The Brunauer–Emmett –Teller (BET) analysis was carried out by AimSizer (AS-3012) equipment to investigate the specific surface area and pore volume of Y_2_O_3_. The self-release behavior of the loaded product (Y_2_O_3_/IMD) was studied by UV-Vis spectroscopy analysis (LAMBDA 650 UV−Vis Spectrophotometer, PerkinElmer, Waltham, MA, USA). The presence of corrosion inhibitor on the surface of steel substrate was further analyzed by XPS (AXIX Ultra DLD, Kratos, UK) using a monochromatic X-ray source—Al Kα source; the reference binding energy of C 1 s was (284.6 eV). XPS wide spectra were recorded in the binding energy range of 250 to 750 eV to determine the elemental composition. High-resolution spectra were recorded at an energy step size of 0.1 eV using a pass energy of 10 eV.

The coating thickness of the coated samples was measured with a gauge meter (PosiTector 6000) and also by FE-SEM observation of the cross section. The adhesion strength of the coated was determined by peel-off test conducted by way of precision adhesion testing equipment (PAT AT101E/6.3 kN, Made in Norway).

The corrosion resistance of the coated samples was studied by electrochemical impedance spectroscopy (EIS) by using a three-electrode electrochemical cell in the 3.5 wt.% NaCl solution. Coated steel sample as working electrode, graphite as counter working electrode and Ag/AgCl as a reference electrode, respectively. The Gamry 3000 (30 K BOOSTER potentiostat/Galvanstate/ZRA, Warminster, PA, USA) was used for EIS analysis within a frequency range of 100 kHz to 10 mHz, which starts from the higher to the lower limit. The EIS was measured at (OCP) for 1 h and the RMS signal was 10 mV.

## 3. Results and Discussion

### 3.1. Structural and Morphological Characterization

**Figure 2:**
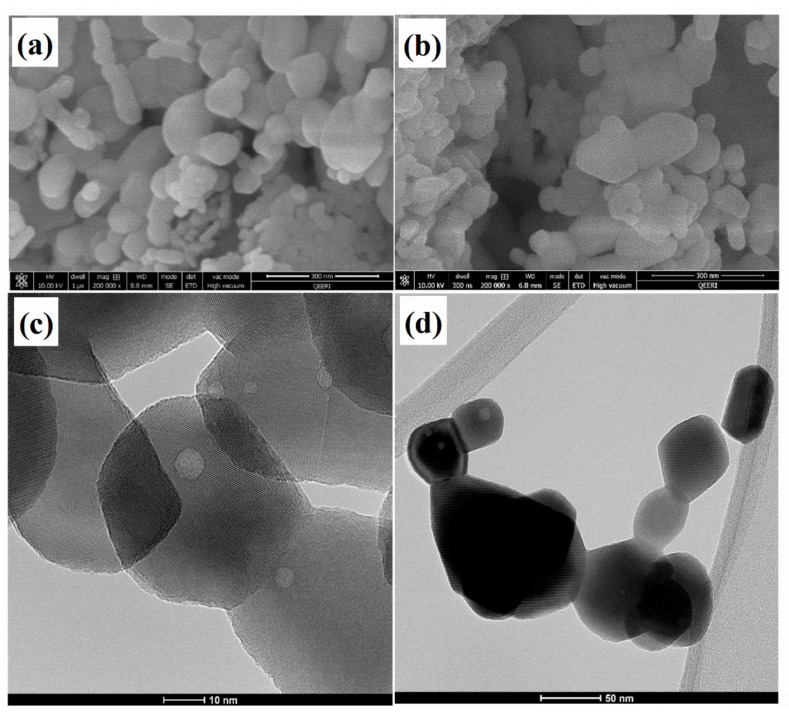
(**a**,**b**) FE-SEM, (**c**,**d**) TEM images of Y_2_O_3_ and Y_2_O_3_/IMD.

The morphological analysis of Y_2_O_3_ and Y_2_O_3_/IMD was investigated FE-SEM and transmission electron microscopy (TEM) and the results are presented in Figure 2. Figure 2a,b evidence a nano textured irregular morphology, with some Y_2_O_3_ particles’ agglomerates that are preserved after loading with the corrosion inhibitor. The size of the particles varies from few tens of nm up to 200 nm for the larger agglomerates. The TEM images shown in Figure 2c indicates that Y_2_O_3_ nanoparticles have some porosity, and its average size is below 50 nm. The darker areas evidenced in Figure 2d suggests the loading of Y_2_O_3_ with imidazole. This evidence was also supported by EDS analysis depicted in (Figure S2a,b) that show the presence of yttrium at 2 keV. The peaks at 0.15 KeV and 0.27 KeV correspond to C and N, respectively. The increase in carbon intensity and the presence of nitrogen confirms the loading of imidazole into the porous Y_2_O_3_ nanoparticles.

The surface charge of the particles was measured by Zeta potential to confirm the loading of imidazole. The mean values of Zeta potential for Y_2_O_3_ are 12.9 mV and −47.89 mV for imidazole presented in Figure S3. The opposite charges of Y_2_O_3_ and imidazole help on the loading process. The mean zeta potential of loaded product Y_2_O_3_/IMD is 2.24 mV which is lower than the value determined for Y_2_O_3_. This also suggests that IMD is present in the porous structure of Y_2_O_3_.

**Figure 3:**
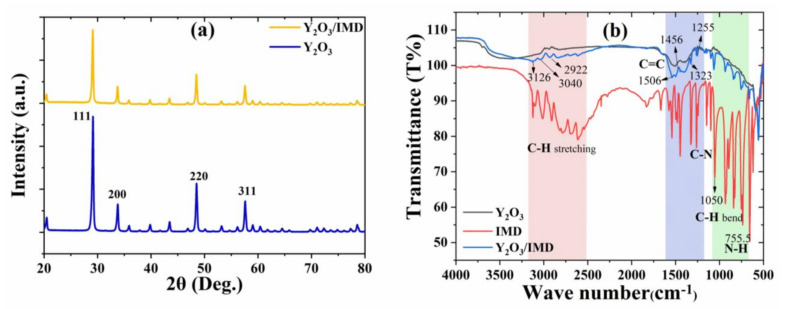
(**a**) XRD and (**b**) FTIR analysis of Y_2_O_3_ and Y_2_O_3_/IMD.

The XRD pattern of Y_2_O_3_ and Y_2_O_3_/IMD are shown in Figure 3a and evidence the particles nature is crystalline. The distinct peaks at 29.11°, 33.81°, 48.45° and 57.61° could be attributed to crystal planes 111, 200, 220, 311 respectively, which is in accordance with JCPDS No: 98-018-5295 of Y_2_O_3_ crystal [23]. No significant differences are observed in the XRD pattern of Y_2_O_3_ and Y_2_O_3_/IMD, and the lack of any extra peak(s) shows no relevant structural or phase changes occurs in the Y_2_O_3_ due to the presence of imidazole.

FTIR spectra of yttrium oxide nanoparticles, IMD and imidazole loaded yttrium oxide nanoparticles are presented in Figure 3b. A strong and sharp peak around 565 cm^−1^ is due to the characteristic of Y-O vibration [27]. The characteristic bands of the two spectra by comparative analysis indicates that loaded Y_2_O_3_ nanoparticles are allied with the stretching vibrations of C-H at around 3126 cm^−1^, 3040 cm^−1^ and 2922 cm^−1^ [28]. As compared to the Y_2_O_3_, the spectra for the loaded Y_2_O_3_ nanoparticles showed the N-H deformation band at around 755.5 cm^−1^ and the C-H bending was observed around 1050 cm^−1^ and 939.6 cm^−1^. The presence of a band at 755 cm^−1^ and 1000 cm^−1^ can be assigned to the NH_2_ bending and C-H bending, respectively, as per literature [29]. According to the spectra for loaded nanoparticles, the appearance of two new bands at about 1323 cm^−1^, 1255 cm^−1^ corresponds to medium–weak C-N stretching in Imidazole [30]. The absorption bands observed at 1506 cm^−1^ and 1456.2 cm^−1^ correspond to the C=C spectrum and evidence the presence of imidazole into the porous Y_2_O_3_ nanoparticles.

#### Additional Physico-Chemical Characterization

**Figure 4:**
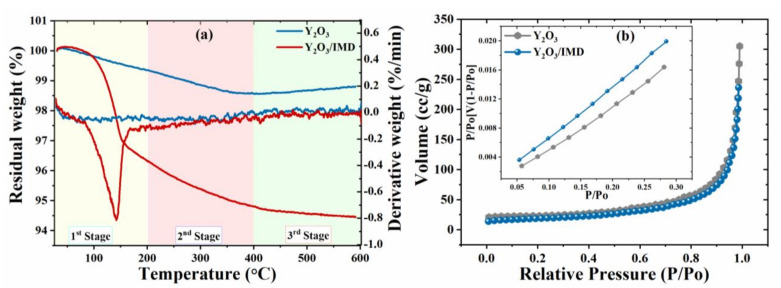
(**a**) TGA and DTA, (**b**) B.E.T analysis of Y_2_O_3_ and Y_2_O_3_/imidazole.

Thermal stability of pristine and loaded yttrium oxide nanoparticles was assessed by TGA. The 1 wt.% weight loss of Y_2_O_3_ nanoparticles is due to removal of absorbed water molecules [27], as shown in Figure 4a. In contrast, weight loss of Y_2_O_3_/IMD observed in three stages of TGA spectra. In the first stage, a sharp weight loss occurs from room temperature (28 °C) to 200 °C, due to the imidazole decomposition [31]. In the second stage (201 °C to 400 °C), weight losses occur due to removing some residual compounds of imidazole until its complete decomposition. While considering that 256 °C is the boiling point of imidazole. In the last stage, between 401 °C to 600 °C, weight loss rate is constant. The pure yttrium oxide nanoparticles remain stable, and no significant weight loss observed. Hence, the weight loss in the case of loaded Y_2_O_3_ nanoparticles can be attributes to the removal of imidazole. It can be inferred from Figure 4a, based on the quantity of the weight loss, approximately 5 wt. % of imidazole were loaded into the porous yttrium oxide nanoparticles. The degradation peak for DTA around 150 °C is due to decomposition of imidazole.

The changes in the cumulative pore volume and specific surface area (SSA) for Y_2_O_3_ and Y_2_O_3_/imidazole are observed by Brunauer–Emmett–Teller (B.E.T.) analysis—Figure 4b. The SSA value for Y_2_O_3_ is 73.729 m^2^g^−1^ before loading, but it is reduced to 62.671 m^2^g^−1^ after loading with imidazole. Similar to SSA, the cumulative pore volume reduced from 0.4744 ccg^−1^ to 0.3675 ccg^−1^ after loading. The reduction in SSA and cumulative pore volume confirms the loading of imidazole into Y_2_O_3_.

**Figure 5:**
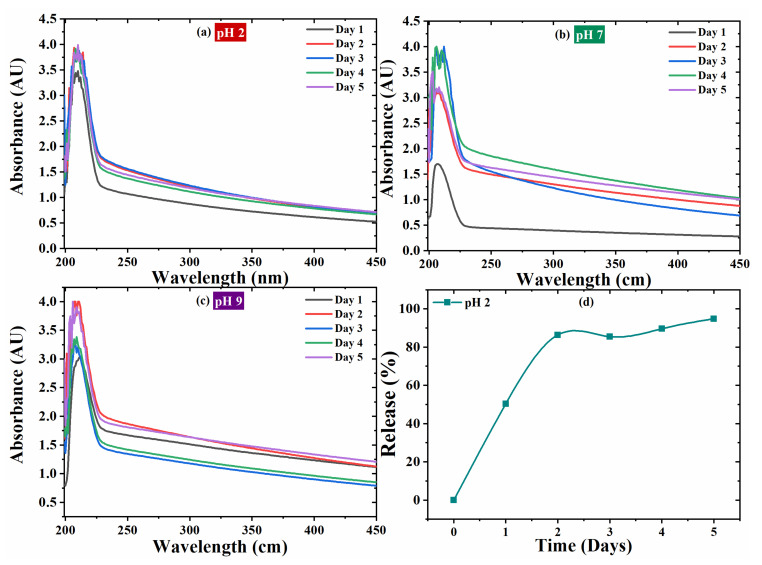
UV-Vis Spectra at (**a**) pH 2, (**b**) 7 and (**c**) 9 for Y_2_O_3_/imidazole, (**d**) release (%) plot.

The UV-Vis spectroscopy analysis evidences the self-release behavior of the imidazole. Thus, 3.5 wt.% of NaCl solutions at different pH values (2, 7 and 9) were prepared and the loaded product (Y_2_O_3_/imidazole) was dispersed in it. The UV-Vis spectra of Y_2_O_3_/imidazole were taken after five days of immersion and presented in Figure 5. The UV spectra profiles are identical at different pH values, and a sharp peak at wavelength of 206 nm can be assigned to the aromatic structure of imidazole [28]. Absorbance peaks can be observed for all pH values (2, 7 and 11). The UV spectra at pH 2 is presented in Figure 5a. The absorbance intensity of 3.5 AU was measured at first day of immersion which slightly increases up to ~3.9 AU and remains constant till Day 5. The absorbance intensity at the first day of immersion is 1.5 AU for pH 7—Figure 5b and increases up to 3.0 AU for the second day and 3.8 AU for the third day. The intensity decreases to 3.0 AU at the fifth day of immersion. The UV spectra taken at pH 11 is presented in Figure 5c and its absorbance intensity increases from 3.0 AU (Day 1) to 4.0 AU (Day 2). After that the intensity decreases to 3.3 AU after three to four days and then increases to 4.0 AU after the fifth day of immersion. A comparative trend with respect to immersion times at different pH values (2, 7 and 11) is presented in Figure 5d. The presence of inhibitor is confirmed in all different pH conditions (acidic, neutral, and basic) but it shows increased absorbance intensity in acidic medium as compared to basic and neutral [31].

**Figure 6:**
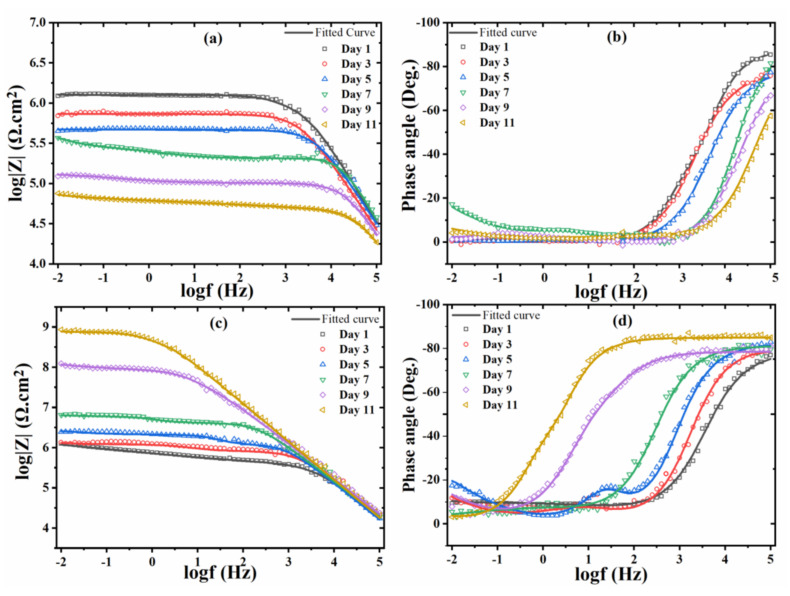
EIS Bode plots of (**a**,**b**) reference coating and (**c**,**d**) Y_2_O_3_/IMD modified coating.

The standard solution curve equation (Supplementary Figure S4) was used to evaluate the release percentage of imidazole from the Y_2_O_3_ and depicted in Figure 6. The release percentage (%) of the corrosion inhibitor from the nano particles was calculated from Equation (1).

Release (%) = M_t_/M_o_ × 100 (1)

where M_t_ is the amount of inhibitor released with time obtained in different pH with UV-Vis spectroscopy and IMD standard curve and M_o_ is the loaded amount from IMD into Y_2_O_3_ determined from TGA results.

### 3.2. Corrosion Resistance of Coated Steel Samples

#### 3.2.1. Electrochemical Impedance Spectroscopy (EIS)

The corrosion resistance of the coated samples was analyzed by electrochemical impedance spectroscopy (EIS) while immersed in NaCl solution. To accelerate corrosion a defect, made with a scalpel, was created in all coated samples. The Bode plots for the reference (only Y_2_O_3_) and modified (Y_2_O_3_/IMD) coatings are shown in Figure 6 and goodness of fit value presented in (Supplementary Table S1). The EIS spectra obtained for the reference coating shows a decrease in the overall impedance values over time, due to the corrosion propagation and electrolyte uptake from the defect made in the coating. The attenuation of the capacitive response in the region of high frequency indicates an increase of the active surface area, and therefore, delamination of the coating [32]. The impedance values for reference coating decrease more than one order of magnitude after 11 days of immersion.

The impedance spectra of the Y_2_O_3_/IMD modified coating show an inverse trend—Figure 6. Initially, the impedance values slightly increase with time. After seven days, the impedance values increased by one order of magnitude and from the seventh to the eleventh days, impedance values increased by two orders of magnitude. The initial increase was slower because as the electrolyte reached the steel substrate, corrosion is likely to start, inducing local pH changes that help to release the corrosion inhibitor. The inhibitor is expected to form a protective layer where the steel surface is exposed [33] that decreases the active surface area, delaying coating delamination and, therefore, increasing impedance values. The increase in impedance for the modified coating is followed by broadening of the high frequency time constant and development of a well-defined capacitive behavior in the region of high frequency [34]. This trend shows that the corrosion activity in and around the surface defect was inhibited and that the area of exposed steel was probably reduced.

**Figure 7:**
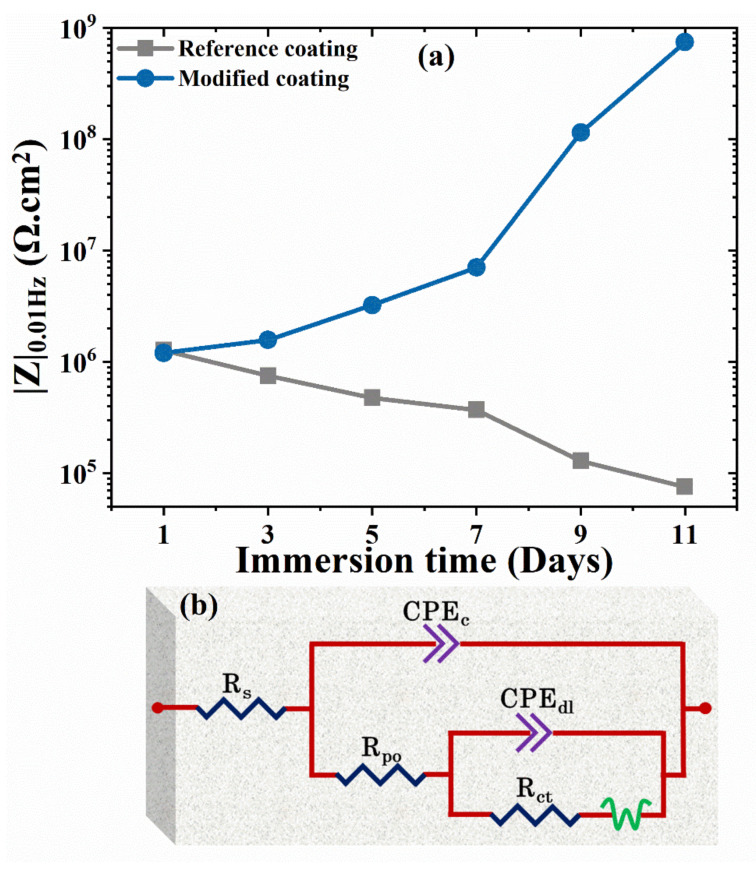
(**a**) Low impedance values for reference and modified coating; (**b**) equivalent circuit.

The low-frequency impedance values for the modified coating increases over time as depicted in Figure 7a. While Nyquist plots for reference and modified coating presented in the Supplementary Data (Figure S6).

**Figure 8:**
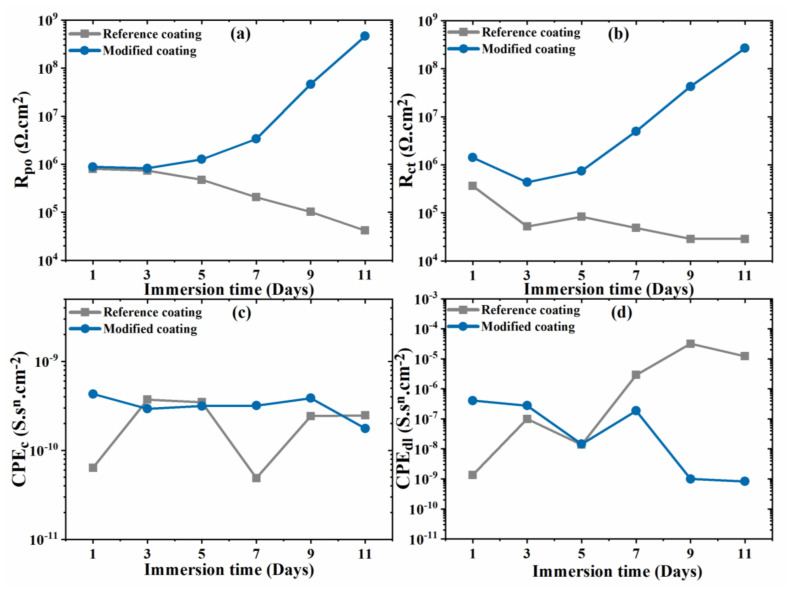
(**a**) Evolution of the pore resistance (R_po_), (**b**) Faradic resistance (R_ct_), (**c**) admittance of coating (CPE_c_), (**d**) admittance of the double layer (CPE_dl_).

The values of relevant EIS parameters, namely solution resistance inside pores and scratch (R_po_), faradic resistance (R_ct_), and respective constant phase elements (CPE_c_, CPE_dl_) were obtained after fitting with the equivalent circuit provided in Figure 7b and Table S1. The high frequency capacitive slope corresponds to the coated areas and its constant phase element, while the low frequency time constant, R_ct_ and CPE_dl_, can be assigned to the surface activity in the scratched areas and bottom of the pores. The EIS fitting values are depicted in Figure 8.

The high frequency resistance (R_po_) of the reference coating decreases from ~1 MΩ.cm^2^ to 41.79 KΩ.cm^2^ after immersion of 11 days—Figure 8a—are due to continuous propagation of the delaminated area from the defect originally formed in the coating. The inhibited coating successfully resists to the progress of delamination probably due to development of a protective layer on the exposed surface of steel which decreases the fraction of metal exposed to the electrolyte [35] and inhibits the corrosion activity attenuating local pH changes, namely alkalization, which is the main responsible for cathodic delamination.

The evolution of the faradic resistance (R_ct_) with time—Figure 8b shows a decrease for the reference coating, for more than one order of magnitude, evidencing that corrosion activity is increasing. Contrarily, for the for the inhibited coating (Y_2_O_3_/IMD) the faradic resistance increased by more than two orders of magnitude, suggesting that the surface activity is hindered in the presence of the particles loaded with the inhibitor.

The CPE values for both coatings are depicted in Figure 8c,d. The high frequency CPE for the reference coating tends to increase revealing increased electrolyte uptake in the coated areas while for the inhibited coating the values are nearly constant, suggesting that the coated parts remain more resistive. The low-frequency admittance values of the constant phase element for the reference coating increases for more than two orders of magnitude with time, probably due to a larger active surface area and progress of coating delamination from the scratch. However, for the inhibited coating, the values tend to be constant/slightly decreasing suggesting that the active surface area decreases, and that delamination was hindered and protecting the bare steel [36].

The impedance values for the reference (Y_2_O_3_) and inhibited coatings (Y_2_O_3_/IMD) at different immersion times were used to calculate the inhibition efficiency (I.E %) [31]—Table 1.

I.E (%) = (1 − R_ct_/R_ct1_) × 100 (2)

where R_ct_ is the faradic resistance value without inhibitor and R_ct1_ is for inhibited coating (Y_2_O_3_/IMD).

#### 3.2.2. XPS Analysis

**Figure 9:**
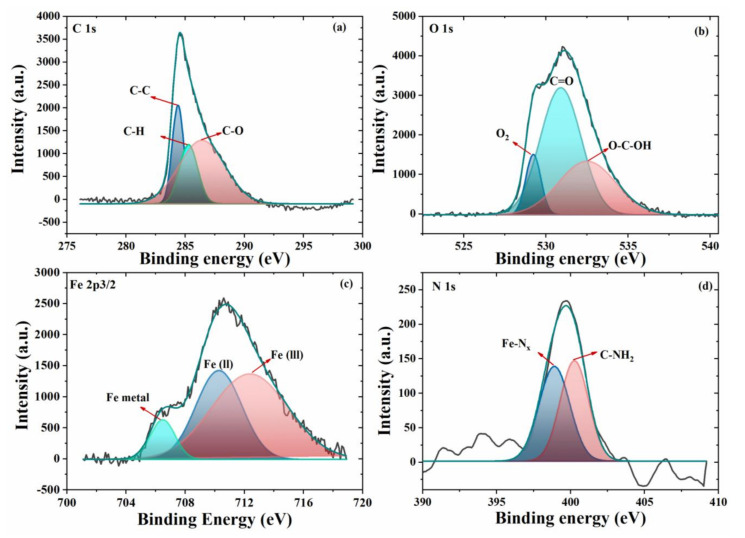
XPS analysis of surface after coating removal**,** High resolution spectra of (**a**) C 1s, (**b**) O 1s, (**c**) Fe 2p^3/2^, (**d**) N 1s.

The EIS results confirms the enhanced protective effect of the modified coating which may be due to formation of a stable protective layer on the metal surface. Thus, it is also important to verify if the adsorption of imidazole on the steel substrate forms a protective film. The coating was removed, and the steel substrate surface was studied by X-ray photoelectron spectroscopy (XPS) to investigate the presence of a protective film. The high resolution XPS spectra for C1s depicted in Figure 9a shows three different peaks. The broad and asymmetric tail towards high binding energy is due the sp^2^ hybridization present in imidazole. The binding energy peak at 284.4 eV and 285.3 corresponds to the C-C and C-H bonding between the epoxy because some epoxy residues might remain on the steel substrate [37]. The peak at 286.4 eV corresponds to carbon and oxygen containing species [38]. The HR-XPS spectra for O 1s can be decomposed into three peaks—Figure 9b. The binding energy at 529.2 eV corresponds to the formation of oxides (likely Fe oxides). The peak at 530.9 eV is due to the presence of C-O bonds, the peak at 532.6 eV corresponds to the presence of hydroxyl species, probably hydrated Fe compounds, [39,40]. The Fe 2p^3/2^ XPS spectra depicted in Figure 9c, reveals a peak at 706.5 eV that can be assigned to the metallic iron. The peaks at 710.3 eV and 712.4 eV corresponds to the Fe (II) and Fe (III) species, probably oxides and hydroxides which can also be observed in XPS spectra for O 1s [41]. The N 1s XPS spectra shown in Figure 9d shows the two peaks. The peak at ~398.6 eV corresponds to the nitrogen species bounded to iron to form a linkage of FeN_x_ [42]. The N1s spectra favors the adsorption of nitrogen on steel surface confirmed by the binding energy peak of FeN_x_, which are consistent with previous study where mostly metal nitride forms in this energy band range [43]. While there is a peak at 400 eV, which is due to the bonding of N-CH_2_. The XPS elemental atomic percentage chemical composition of the surface is provided in the Table 2. The presence of nitrogen confirms the adsorption of imidazole on the steel substrate.

**Figure 10:**
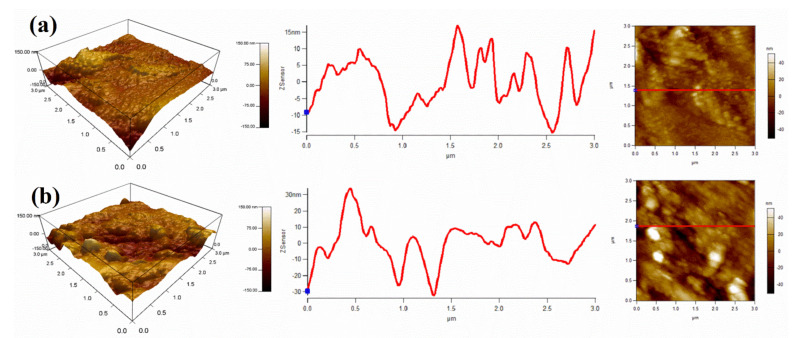
AFM analysis of (**a**) steel in absence of IMD, and (**b**) steel substrate in the presence of IMD.

The surface roughness of the surface after coating removal was measured by atomic force microscopy (AFM)—Figure 10b. Results seem to indicate that the steel surface of inhibited coating at the end of the EIS tests is relatively rough compared to the surface of reference coating. The surface roughness (R.M.S.) for the steel substrate of reference coating is R.M.S. = 9.542 nm, that is increased to 15.937 nm in the presence of IMD (Figure 10). The increment in the surface roughness value can ascribed to the development of a thicker protective layer on the steel substrate surface in the presence of IMD.

### 3.3. Corrosion Inhibition Mechanism

**Figure 11:**
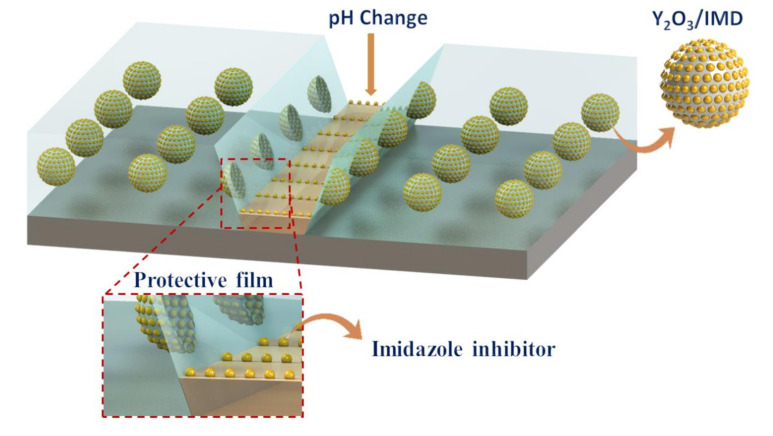
The schematic diagram explaining inhibition mechanism.

The UV-Vis shows that loaded product (Y_2_O_3_/IMD) seems to be pH sensitive, a property that helps to release the inhibitor from the particles and improves corrosion inhibition as demonstrated by EIS. Initially when the electrolyte reaches steel the local pH changes due to corrosion activity [44]. The pH at the scratched area is expected to reach acidic values in the anodic areas due to Fe ions hydrolysis aiding on the release of the corrosion inhibitor. The inhibition effect observed for the Y_2_O_3_/IMD—modified coating is due to the adsorption ability of imidazole on the steel substrate, according to the schematic diagram depicted in Figure 11.

The inhibitor, which has been reported as a mixed-type-film forming corrosion inhibitor [45], is able to form a protective layer over steel slow downing corrosion activity. In fact, IMD shows three possible anchoring sites [46]: the nitrogen atom and the sp2 lone pair of electrons, the active hydrogen bon attached to the N atom and the π bonds that are characteristic of the aromatic ring. The formation of a stable layer containing the inhibitor, which has been reported as hydrophobic, repeals water and aggressive species [47] and is responsible for the improved corrosion resistance. Possible reactions between the inhibitor and epoxy, contributing to higher coating resistance, can also be present and cannot be discarded [48,49]. Advanced modeling calculations [50] have shown that the stable inhibition performance of imidazole is due to the formation of a surface layer in which a cooperative effect between adsorption due to breaking of the double bonds in the aromatic rings and the formation of C-C sigma bonds between different molecules of the inhibitor. Interestingly, as the number of possible C-C sigma intermolecular bonds increases, the more stable and protective the surface film is. This “polymerization”-like effect is, therefore, responsible for the improved corrosion protection of the steel surface as demonstrated by EIS.

## 4. Conclusions

The anti-corrosive pigments Y_2_O_3_/Imidazole were synthesized and dispersed in an epoxy formulation to prepare modified coatings for corrosion protection of steel substrates. The loading of imidazole into Y_2_O_3_confirmed by FTIR, while TGA evidences the loading capacity of the Y_2_O_3_ nanoparticles. EIS studies reveal that the modified coating shows better anti-corrosive properties compared to the reference coating, probably due to a positive interaction between the inhibitor and the epoxy-based matrix. The EIS parameters show an increase of the corrosion activity in the non-inhibited coating and an important protection effect in the presence of the Y_2_O_3_/imidazole modified coating. The imidazole release from the Y_2_O_3_ nanoparticles might be related to local changes in pH and formation of a stable and more protective layer over the exposed steel substrate. XPS analysis of the surface beneath the coating confirmed the presence of imidazole at the coating-metal interface at the end of the immersion tests in NaCl.

## Figures and Tables

**Table 1:** Faradic resistance and corrosion inhibition efficiency of reference and Y_2_O_3_/IMD- modified coatings with change in immersion time.

Immersion Time (Days)	Faradic Resistance (R_ct_ (Ω.cm^2^)		
	Reference Coating	Modified Coating	I.E (%)
1	3.630 ± 0.7 × 105	1.411 ± 0.6 × 106	-
3	5.211 ± 0.7 × 104	4.344 ± 0.4 × 105	16.5
5	8.311 ± 1.2 × 104	7.475 ± 1.4 × 105	51.4
7	4.855 ± 1.5 × 104	4.966 ± 1.1 × 106	92.6
9	2.859 ± 0.2 × 104	4.264 ± 0.3 × 107	99.1
11	2.856 ±0.1 × 104	2.678 ± 0.2 × 108	99.8

**Table 2:** Surface elemental composition of surface of XPS data.

Sample	Surface Chemical Composition, Atomic %			
	C	O	N	Fe
Modified coating surface	56.9	25.6	2.9	55.8

## Data Availability

The raw data required to reproduce these findings cannot be shared at this time as the data also forms part of an ongoing study.

## Supplementary Materials

The following are available online at https://www.mdpi.com/article/10.3390/nano11092291/s1, Figure S1: Cross sectional image of dried coating thickness, Figure S2: EDS analysis of (a) Y_2_O_3_ and (b) Y_2_O_3_/IMD, Figure S3: Zeta potential of Y_2_O_3_ and Y_2_O_3_/Imidazole, Figure S4: (a) standard solution curve (b) Release percentage at pH 2, 7 and 9, Figure S5: Statistical distribution data of impedance value for (a) Reference (b) Modified coating, Figure S6: Nyquist plot (a) Reference and (b) Modified coatings, Table S1: EIS fitting parameters value for reference (Y_2_O_3_) and modified coating (Y_2_O_3_/IMD).
