# Supplementary material for: Effectiveness of Epoxy Coating Modified with Yttrium Oxide Loaded with Imidazole on the Corrosion Protection of Steel
Source: Nanomaterials (Basel). 2021 Sep 3;11(9):2291. doi: 10.3390/nano11092291 (PMC8469726; doi:10.3390/nano11092291)
Supplement: Supplementary file 1 [file nanomaterials-11-02291-s001.zip › nanomaterials-1332433-supplementary.pdf]

nanomaterials

Supplementary Materials

Effectiveness of Epoxy Coating Modified with Yttrium Oxide Loaded with Imidazole on the Corrosion Protection of Steel

Muddasir Nawaz [1], Nazal Naeem [1], Ramazan Kahraman [2,*], M. F. Montemor [3], W. Haider [1] and R. A. Shakoor [1,*]

1 Center for Advanced Materials (CAM), Qatar University, Doha 2713, Qatar; m.nawaz@qu.edu.qa (M.N.); nazalnaeem14@gmail.com (N.N.); haide1w@cmich.edu (W.H.)
2 Department of Chemical Engineering, Qatar University, Doha 2713, Qatar
3 Centro de Química Estrutural, Departamento de Engenharia Química, Instituto Superior Técnico, Universidade de Lisboa, Av Rovisco Pais, 1049-001 Lisboa, Portugal; mfmontemor@tecnico.ulisboa.pt
4 Mechanical and Materials Engineering, Central Michigan University, Mount Pleasant, MI 48859 USA
* Correspondence: ramazank@qu.edu.qa (R.K.); shakoor@qu.edu.qa (R.A.S.); Tel: +974-44034130 (R.K.); +974-44036867 (R.A.S.)

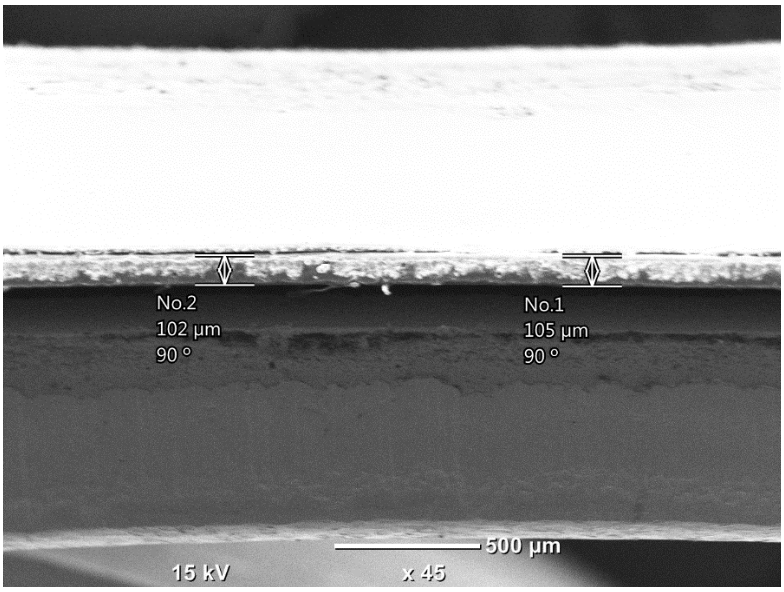

Figure S1. Cross sectional image of dried coating thickness.

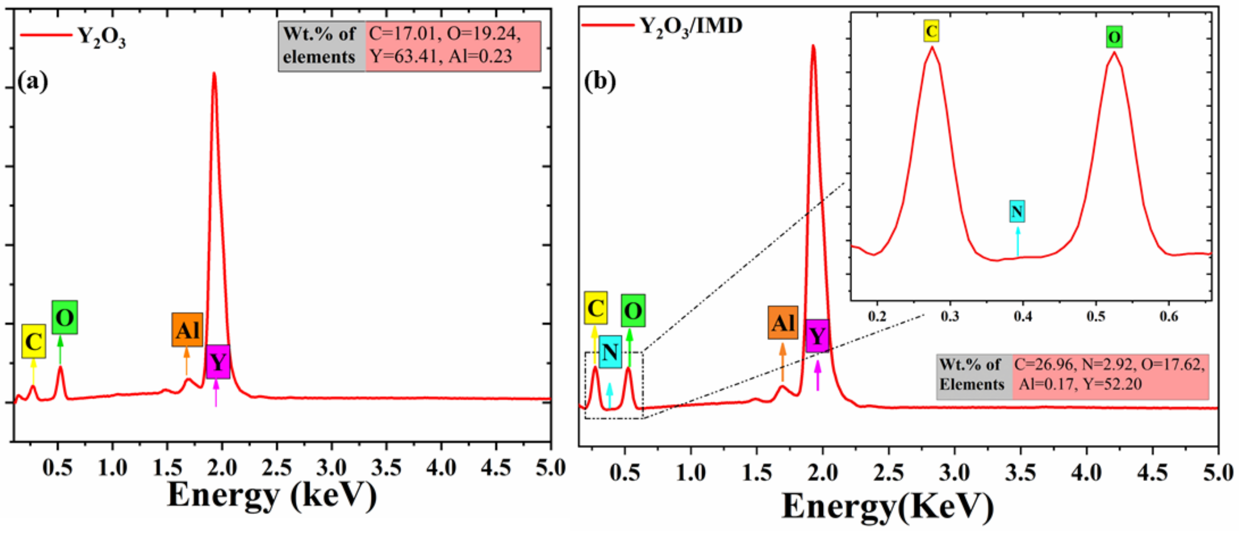

Figure S2. EDS analysis of (**a**) Y_2O_3 and (**b**) Y_2O_3/IMD.

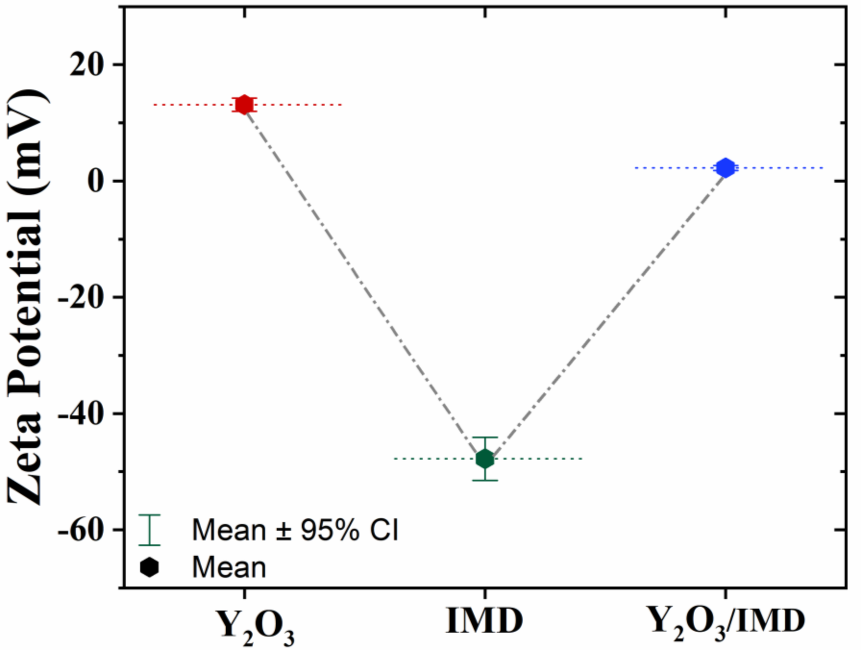

Figure S3. Zeta potential of Y_2O_3 and Y_2O_3/Imidazole.

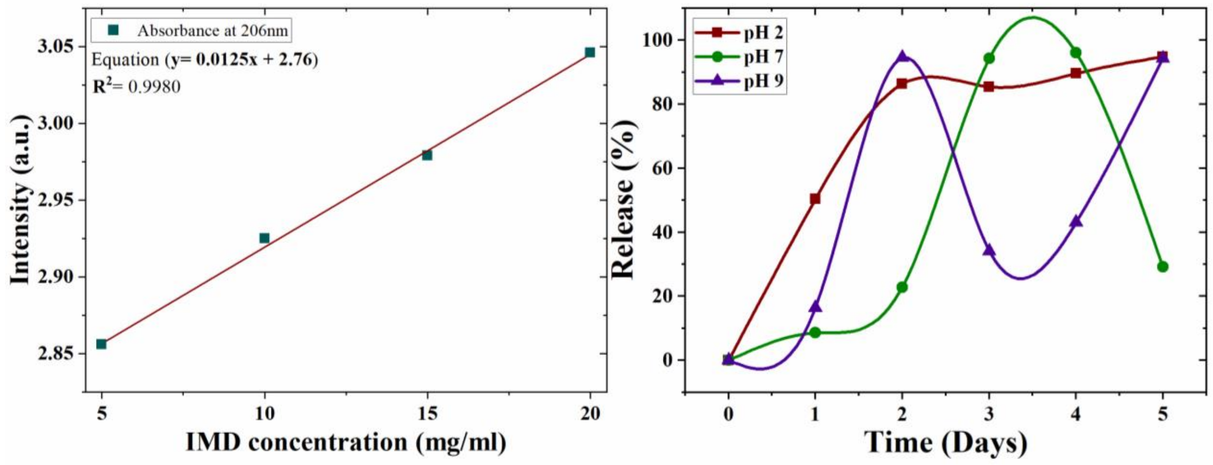

Figure S4. (**a**) standard solution curve (**b**) Release percentage at pH 2, 7 and 9.

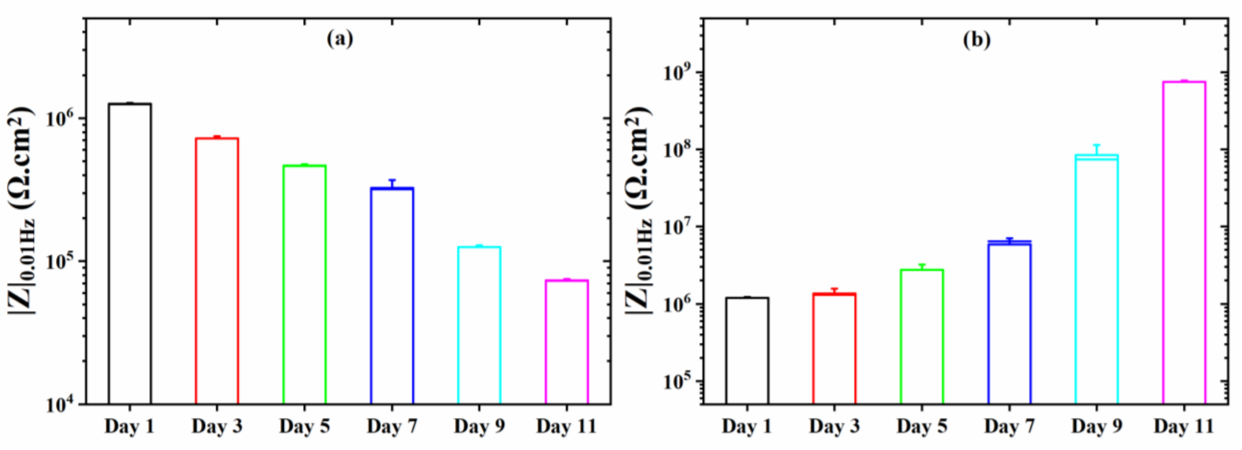

Figure S5. Statistical distribution data of impedance value for (**a**) Reference (**b**) Modified coating.

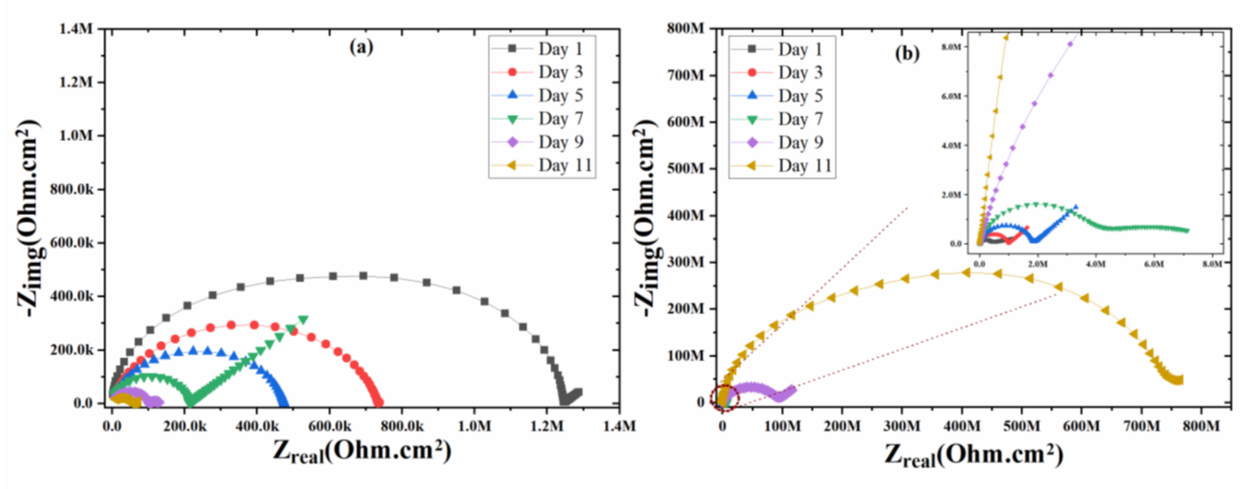

Figure S6. Nyquist plot (**a**) Reference and (**b**) Modified coatings.

Table S1. EIS fitting parameters value for reference (Y_2O_3) and modified coating (Y_2O_3/IMD).

Coatings	Day	R_{ct} (Ω.cm²)	CPE_c ($S.s^n.cm^2$)	n	R_{po} (Ω.cm²)	CPE_{dl} ($S.s^n.cm^2$)	n	Goodness of Fit
Y_2O_3	1	$3.630x10^5$	$6.368x10^{-11}$	0.977	$8.019x10^5$	$1.351x10^{-9}$	0.984	$38.58x10^{-3}$
	3	$5.211x10^4$	$3.715x10^{-10}$	0.864	$7.340x10^5$	$9.914x10^{-8}$	0.734	$138.3x10^{-3}$
	5	$8.311x10^4$	$3.482x10^{-10}$	0.876	$4.747x10^5$	$1.376x10^{-8}$	0.612	$35.73x10^{-3}$
	7	$4.855x10^4$	$4.887x10^{-11}$	0.991	$2.065x10^5$	$2.901x10^{-6}$	0.713	$315.1x10^{-3}$
	9	$2.859x10^4$	$2.42.4x10^{-10}$	0.899	$1.019x10^5$	$3.172x10^{-5}$	0.566	$748.5x10^{-6}$
	11	$2.856x10^4$	$2.474x10^{-10}$	0.908	$4.179x10^4$	$1.212x10^{-5}$	0.318	$232.4x10^{-6}$
Y_2O_3/Imidaz-ole	1	$1.411x10^6$	$4.299x10^{-10}$	0.870	$8.819x10^5$	$4.065x10^{-7}$	0.796	$6.629x10^{-3}$
	3	$4.344x10^5$	$2.936x10^{-10}$	0.897	$8.198x10^5$	$2.748x10^{-7}$	0.630	$28.7x10^{-3}$
	5	$7.475x10^5$	$3.142x10^{-10}$	0.912	$1.263x10^6$	$1.434x10^{-8}$	0.961	$57.65x10^{-3}$
	7	$4.966x10^6$	$3.176x10^{-10}$	0.902	$3.372x10^6$	$1.856x10^{-7}$	0.338	$182.4x10^{-3}$
	9	$4.264x10^7$	$3.866x10^{-10}$	0.872	$4.615x10^7$	$9.958x10^{-10}$	0.756	$102.5x10^{-3}$
	11	$2.678x10^8$	$1.761x10^{-10}$	0.946	$4.613x10^8$	$8.229x10^{-10}$	0.943	$285.1x10^{-3}$
